# Factors associated with cognitive impairment during the first year of treatment for nonmetastatic breast cancer

**DOI:** 10.1002/cam4.3715

**Published:** 2021-01-16

**Authors:** Nicole Rodriguez, Jonathan M. Fawcett, Joshua A. Rash, Renee Lester, Erin Powell, Connor D. MacMillan, Sheila N. Garland

**Affiliations:** ^1^ Department of Psychology Faculty of Science Memorial University St. John’s Newfoundland Canada; ^2^ Discipline of Oncology Faculty of Medicine Memorial University St. John’s Newfoundland Canada

**Keywords:** breast cancer, cognitive impairment, perceived cognitive impairment, insomnia, fatigue, mood disturbance

## Abstract

**Background:**

Women with breast cancer are more likely to develop cognitive impairment (CI), insomnia, fatigue, and mood disturbance than individuals with other cancers. The main objectives of this study were to establish the prevalence of CI and examine the relationships between CI, insomnia, fatigue, and mood over the first year of breast cancer treatment.

**Methods:**

Participants were recruited after diagnosis and completed validated measures of insomnia, objective and perceived CI, fatigue, and mood disturbance at four time points during the first year of treatment. A random intercepts cross‐lagged panel model assessed relationships among symptoms over time.

**Results:**

The sample included 98 women. Prevalence of objective CI ranged from 3.1% to 8.2% throughout the year, whereas 36.7% demonstrated a clinically meaningful decline in perceived CI from baseline to 4 months, which remained relatively stable. Greater perceived CI was associated with more fatigue (*β* = −0.78, *z* = 17.48, *p* < .01) and symptoms of insomnia (*β* = −0.58, *z* = 5.24, *p* < .01). Short‐term fluctuations in perceived CI (*p* < .05), but not fatigue or insomnia, predicted future perceived CI. Fatigue (*p* < .001) was a significant predictor of future reported symptoms of fatigue and insomnia.

**Conclusion:**

Subjective CI is more prevalent than objective impairments. Fatigue, insomnia, and perceived CI remain stable and are associated during the first year of treatment. Changes in insomnia and fatigue may have little effect on future perceived cognition. Women with breast cancer likely require targeted intervention for these side effects.

## Graphical abstract

This study recruited 98 women with non metastatic breast cancer shortly after diagnosis and assessed their objective and perceived cognitive function alongside their levels of insomnia, fatigue, and mood disturbance at 4 time points: shortly after diagnosis and 4 months, 8 months, and 12 months later. Subjective impairment was more prevalent than objective impairment and both remained stable across the year. Perceived impairment in cognition was associated with fatigue and insomnia symptoms, suggesting the need for interventions that can address co‐occurring symptoms.

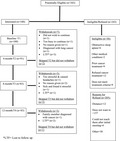

## 1 BACKGROUND

Cognitive impairment (CI) in women with breast cancer typically presents as difficulties with memory, learning, attention, executive function, and/or processing speed.^1^, ^2^ Estimates of the prevalence of CI vary widely as a result of inconsistency in definitions and measurement of CI; however, research suggests that roughly one‐third of breast cancer patients experience CI prior to treatment, which increases to 75 percent during active treatment, and remains at 35 percent in the months and years after treatment completion.^3^

CI seldom occurs in isolation and is typically observed alongside other distressing symptoms, including insomnia, fatigue, and psychological distress.^4^ Women with breast cancer are more likely to develop insomnia,^5^ fatigue,^6^ and mood disturbance^7^ than individuals with other cancers and their noncancer peers. The available research supports an association between CI, insomnia, fatigue, and mood symptoms, although the direction of the relationships has not been elucidated. Cross‐sectional data have illustrated that breast cancer patients with mild, moderate, and severe insomnia symptoms report greater perceived CI than patients without insomnia symptoms.^8^ Studies have also demonstrated a relationship between subjective CI, and fatigue,^9^ anxiety,^10^, ^11^ and depression.^11^ This co‐occurrence suggests these symptoms may result from shared physiological and/or behavioral mechanisms that may together contribute to CI above and beyond the contribution of a single side effect. It is also possible that the presence of one symptom exacerbates others, which further contributes to greater overall symptom burden and worse CI. Prospective studies are needed to explore the relative contribution of insomnia, fatigue, and mood disturbance to CI during the first year of active treatment.

## 2 OBJECTIVE

The first objective of the current study was to characterize the prevalence of objective CI and change in perceived CI among women with breast cancer during the first year of treatment. The second objective was to examine the relationships between perceived CI, insomnia, fatigue, and mood over time. We hypothesized that symptoms of insomnia, fatigue, and mood disturbance at earlier time points would predict higher levels of perceived CI at later time points.

## 3 METHODS

### 3.1 Participants

One‐hundred women with breast cancer were recruited from a regional cancer clinic after receiving their breast cancer diagnosis (i.e., after receiving surgery but prior to beginning adjuvant treatment). Eligibility criteria were: (a) female sex; (b) English‐speaking; (c) over 18‐years of age; (d) a diagnosis of stage I‐III breast cancer; and (e) scheduled to receive adjuvant hormone therapy (i.e., tamoxifen or an aromatase inhibitor), chemotherapy, radiation, or trastuzumab if indicated). Exclusion criteria included: (a) previous treatment for cancer or currently undergoing treatment; (b) presence of a sleep disorder other than insomnia that was not currently managed, such as sleep apnea; (c) presence of a psychological disorder that was not stable and/or would impair the individual's ability to participate in the study, such as schizophrenia; and (d) a score lower than 24 on the Mini Mental State Examination (MMSE; i.e., a score suggestive of severe cognitive impairment).

### 3.2 Procedure

The oncologists screened clinical charts to identify potentially eligible women. Assessments occurred shortly after their clinic visit and after informed consent was obtained. A medical, psychological and sleep disorder screener and the MMSE^12^ were administered during the first assessment to evaluate inclusion and exclusion criteria. Participants were assessed four times over the course of one year: prior to treatment (i.e., T1), and 4‐ (i.e., T2), 8‐ (i.e., T3), and 12‐months after commencing treatment (i.e., T4).

All assessments were completed in person or remotely via telehealth for those located in rural areas. Self‐report measures were mailed to those completing telehealth assessments along with a postage paid return envelope.

### 3.3 Measures

All clinical variables were abstracted from medical charts, and a demographics questionnaire was used to characterize the sample.

#### 3.3.1 Subjective cognitive measures

Perceived cognitive impairment was assessed using the Perceived Cognitive Impairment (PCI) subscale of the Functional Assessment of Cancer Therapy—Cognitive Function (FACT‐Cog), version 3.^13^ Higher scores on the FACT‐cog reflect fewer perceived cognitive problems and better quality of life. A decline of 5.6 points on the PCI subscale represents clinically meaningful perceived declines in cognitive functioning.^14^

#### 3.3.2 Objective cognitive measures

The use of the following objective cognitive measures was based on the recommendations put forth by the International Cognition and Cancer Task Force (ICCTF).^15^ Considering the rural and remote population distribution of Newfoundland and Labrador, only measures that have been validated for administration via telehealth were used.^16^, ^17^, ^18^ The Hopkins Verbal Learning Test‐Revised (HVLT‐R) is a measure of verbal learning and memory, including immediate recall, delayed recall, and delayed recognition.^19^ The Controlled Oral Word Association Test (COWAT) measures verbal fluency and cognitive and motor speed that involves areas of executive functioning such as cognitive flexibility, strategy utilization, suppression of interference, and response inhibition.^20^ Letter‐Number Sequencing (LNS) measures working memory, attention, and cognitive control.

#### 3.3.3 Symptom measures

The Insomnia Severity Index (ISI)^21^ is a seven‐item measure of insomnia severity and higher scores are indicative of more severe insomnia symptoms. The Hospital Anxiety and Depression Scale (HADS) is a 14‐item self‐report measure with subscales that measure anxiety and depression symptoms in the past week.^22^ Higher scores indicate worse mood symptoms. The Multidimensional Fatigue Inventory‐Short Form (MFSI‐SF) is a 30‐item self‐report measure used to assess the various manifestations of fatigue.^23^ Higher scores are reflective of greater levels of fatigue.

### 3.4 Statistical analysis

#### 3.4.1 Missing data analysis

One participant discontinued participation halfway through the first assessment and was removed due to the large proportion of missing data. One participant was identified as a multivariate outlier (Mahalanobis distance exceeding the χ^2^(9) critical of 37.70),^24^ and their data were removed. Little's test for missing completely at random (MCAR) indicated that data were missing completely at random, *χ* ^2^ = 2588.223, *p* > .999. Missing data were singly imputed using estimation‐maximization in SPSS 26.

#### 3.4.2 Prevalence of decline in perceived CI

Difference scores in perceived CI relative to baseline were calculated over time and prevalence of decline in perceived CI was quantified as the proportion of women who experienced a change greater than the recognized cutoff of 5.6 points on the PCI subscale. Lower scores on the FACT‐cog PCI subscale are indicative of worse perceived CI.

#### 3.4.3 Changes in perceived CI, insomnia, fatigue, and mood across time

A series of four (Time: T1, T2, T3, T4) repeated measures analysis of variances (ANOVAs) were conducted with cognition, insomnia, fatigue, and mood as dependent variables to evaluate change across time. Greenhouse‐Geisser corrections were used in cases where sphericity was violated. Significant time effects were followed up with pairwise comparisons using the Bonferroni correction to adjust for inflation in family‐wise error associated with performing multiple statistical tests.

#### 3.4.4 Prevalence of objective CI

Objective CI was defined in concordance with the International Cancer and Cognition Task Force (ICCTF) recommendations of ≥2 standard deviations (SD) *below* published normative means on at least one objective cognitive test or ≥1.5 SD *below* published normative means on two or more objective tests.^15^ Frequencies were tabulated to characterize the prevalence of objective CI at each time point.

#### 3.4.5 Relationship between cognition, insomnia, fatigue, and mood across time

Structural equation modeling was used to examine how perceived CI related to insomnia and fatigue over time. Our original intention was to evaluate symptoms of insomnia, fatigue, and mood; however, mood was excluded from the analysis because of concerns of multicollinearity with fatigue. The inclusion of mood into the model did not appreciably alter results. Cross‐lagged panel models (CLPM) estimate the covariation of multiple variables across time points—while accounting for all other variables in the model—to infer causal influences. We used a Random Intercepts Cross‐Lagged Panel Model (RI‐CLPM) to assess causal pathways between perceived CI, insomnia, and fatigue. The RI‐CLPM breaks down observed variables into two latent components, including trait‐like, time‐invariant, or “between‐person” factors that are controlled for and state‐like, time‐varying, or “within person” factors that are used to estimate autoregressive and cross‐lagged effects for hypothesis testing. We chose the random intercepts variant of the CLPM because our interest was in evaluating the within‐person causal relations between variables after taking between‐person factors that are stable over time into account. For further details on the RI‐CLPM, see Hamaker et al., 2015^25^ and Lim et al., 2016.^26^

Syntax for the RI‐CLPM was generated using the R‐package *riclpmr*,^27^ which was implemented using *lavaan* ^28^ in the *R* programing language.^29^ To simplify, we evaluated a model in which the lagged effects (i.e., cross paths and stability paths) and correlated error terms at 4‐, 8‐, and 12‐months were constrained to be equal over time; constraining our terms in this manner implicitly assumes that the nature and magnitude of the relationship between a given variable at *T*‐*1* and another variable at time *T* is always the same from one time window to the next. Supporting this decision, the more parsimonious constrained model was favored over a model where these parameters were free to vary as evaluated using either the Akaike Information Criterion (ΔAIC = 9.6 in favor of the simpler model) or the Bayesian Information Criterion (ΔBIC = 87.1 in favor of the simpler model). Model fit was evaluated using the Tucker‐Lewis incremental fit index (TLI^30^), and root mean square error of approximation (RMSEA^31^) where values ≥0.90 and ≤0.08 were considered good fit, respectively.^32^

## 4 RESULTS

### 4.1 Demographic & clinical characteristics

**FIGURE 1:**
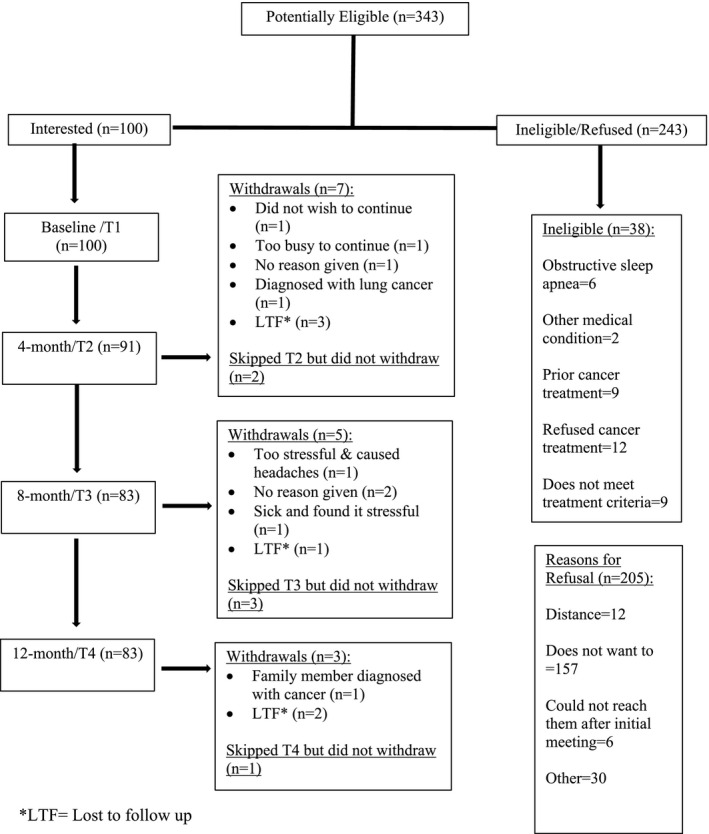
Study flow diagram demonstrating the number of eligible participants and the number of participants who withdrew at each time point. One‐hundred participants completed a baseline assessment. A total of 15 participants dropped out of the study

Three hundred and forty‐three women with breast cancer were approached between January of 2017 and February of 2019; refer to Figure 1 for a study flow diagram. Data from 98 participants were analyzed with an attrition rate of 15%. On average, participants were 60 years of age (range 29–83) and had 13.6 years of education (range 7–25). Refer to Table 1.

**TABLE 1:** Demographic and clinical characteristics

		(*N* = 98) *N* (%)
Age at enrollment (mean ± SD)		60.12 ± 11.05 (range = 29–83)
BMI (mean ± SD)		29.70 ± 7.26 (range = 17.02–51.70)
Marital Status	Married/Committed relationship	70 (71.4%)
	Divorced	7 (7.1%)
	Single	8 (8.2%)
	Widowed	12 (12.2%)
	Other	1 (1.0%)
Number of children	None	8 (8.2%)
	One or two	50 (51.1%)
	Three or more	40 (40.8%)
Race	White/Caucasian	94 (95.9%)
	Other	4 (4.1%)
Education	Some high school (<11 years)	16 (16.3%)
	High school (11 years)	18 (18.4%)
	College (12–14)	28 (28.6%)
	Postsecondary (≥15)	36 (36.7%)
Currently employed	Yes	36 (36.7%)
	No	62 (63.3%)
Premenopausal	Yes	25 (25.5%)
	No	71 (72.4%)
	Unsure	2 (2.0%)
Surgery	Lumpectomy	40 (40.8%)
	Simple mastectomy	48 (49.0%)
	Modified radical mastectomy	10 (10.2%)
	Sentinel node biopsy	74 (75.5%)
	Axillary lymph node dissection	15 (15.3%)
Adjuvant therapy	Chemotherapy	22 (22.4%)
	Radiation	52 (53.1%)
	Trastuzumab	3 (3.6%)
Hormone therapy	Tamoxifen	19 (19.4%)
	Aromatase inhibitor	79 (80.6%)
T Stage	T1	67 (68.4%)
	T2	24 (24.5%)
	T3	6 (6.1%)
	T4	1 (1.0%)
Estrogen receptor positive	Yes	98 (100.0%)
	No	0 (0.0%)
Progesterone receptor positive	Yes	91 (92.9%)
	No	7 (7.1%)
HER2 positive	Yes	5 (5.1%)
	No	93 (94.9%)

Caucasians made up 95.9% of the sample. On average, participants were 60.1 years old (range 29–83) and had 13.6 years of education (range 7–25).

Abbreviations: BMI, Body Mass Index; HER2, human epidermal growth factor receptor 2.

### 4.2 Prevalence of objective CI and changes in perceived CI

Prior to commencing chemotherapy, radiation, and/or hormonal therapies, 6.1% of participants met the ICCTF criteria for objective CI at baseline; 8.2% met criteria at 4 months; 7.1% at 8 months; and 3.1% at 12 months. In contrast, 36.7% of participants demonstrated a clinically meaningful decline in perceived CI from baseline to 4 months and this remained relatively constant throughout the study period. See Table 2. Exploratory comparisons were performed to determine whether those who reported a clinically meaningful decline in perceived CI differed from those who did not report a decline on demographic, clinical, or symptom measures. Individuals with perceived CI at 4 months reported more hours of physical activity at baseline (4.49 ± 4.72) than those without perceived CI (2.76 ± 2.77), *t*(96) = 2.29, *p* = .02, Cohen's *d* = 0.48. Those with greater perceived CI also reported greater insomnia (11.74 vs. 8.64; *p* = .02, Cohen's *d* = 0.50), greater fatigue (22.71 vs. 7.47; *p* = .001, Cohen's *d* = 0.78), and greater depressive symptoms (5.58 vs. 2.98; *p* = .002, Cohen's *d* = 0.73) than those without perceived CI. No other differences were observed between groups on demographic, clinical, or symptom characteristics.

**TABLE 2:** Prevalence of objective CI and changes in perceived CI across time

		(*N* = 98) *N* (%)
Pretreatment assessment (T1)	Objective cognitive impairment	6 (6.1%)
4‐month assessment (T2)	Objective cognitive impairment	8 (8.2%)
	Significant decline in perceived cognition from T1 to T2	36 (36.7%)
8‐month assessment (T3)	Objective cognitive impairment	7 (7.1%)
	Significant decline in perceived cognition from T1 to T3	35 (35.7%)
12‐month assessment (T4)	Objective cognitive impairment	3 (3.1%)
	Significant decline in perceived cognition from T1 to T4	35 (35.7%)

Six participants (6.1%) met criteria for objective cognitive impairment prior to commencing treatment, 8.2% at T2 7.1% at T3, and 3.1% at T4. A significant decline in perceived cognitive functioning was observed in 36.7% of participants from T1 to T2, 35.7% from T2 to T3, and 35.7% from T3 to T4.

Abbreviation: CI, cognitive impairment.

### 4.3 Changes in perceived CI, insomnia, fatigue, and mood

There was a statistically significant difference in perceived CI across the 12 months [*F*(2.629, 255.049) = 8.60, *p* < .001]. Perceived CI scores at baseline were significantly better than perceived CI scores at 4‐, 8‐, and 12‐months. There was a statistically significant difference in insomnia severity between time points [*F*(3, 291) = 11.13, *p* < .001]. Insomnia increased at 4‐months and remained stable across the remaining assessments. Fatigue scores changed significantly over time [*F*(2.337, 226.658) = 7.47, *p* < .001], with increases from baseline observed at both 4‐ and 8‐months. Fatigue scores decreased again at 12‐months but was not significantly different from any other time point. There was a statistically significant difference in depression scores over time [*F*(3, 291) = 4.14, *p* = .007]. Depression scores at baseline were significantly lower than scores at 4‐months. The remaining pairwise comparisons between T2, T3, and T4 were not statistically significant. There was not a statistically significant difference in anxiety symptoms over time [*F*(3, 291) = 0.338, *p* = .798]. See Table 3.

**TABLE 3:** One‐way ANOVAS demonstrating changes in PCI, insomnia, fatigue, and mood across time

		T1	T2	T3	T4	*F*	*p*
PCI^c^	Mean	56.45	**52.96** ^b^	**52.68** ^b^	**52.62** ^b^	8.60	.**000**
	SD	10.09	10.65	10.73	10.79		
Insomnia	Mean	6.81	**9.91** ^b^	**9.24** ^b^	**8.48** ^a^	11.13	.**000**
	SD	6.03	6.10	6.04	6.08		
Fatigue	Mean	5.84	**12.77** ^b^	**13.10** ^b^	10.03	7.47	.**000**
	SD	17.95	19.85	18.56	17.31		
Depression	Mean	3.04	3.97*	3.64	3.34	4.14	.**007**
	SD	3.12	3.61	2.99	2.85		
Anxiety	Mean	5.85	5.88	5.98	5.66	.338	.798
	SD	4.07	3.70	3.60	3.51		

A significant difference in scores was found in perceived cognitive impairment (*p* = .000) from T1 to T2, T1 to T3, and T1 to T4; a significant difference in scores was found in insomnia (*p* = .000) from T1 to T2, T1 to T3, and T1 to T4; a significant difference was found in fatigue scores (*p* = .000) from T1 to T2 and T1 to T3; a significant difference was found in depression scores (*p* = .007) from T1 to T2; no significant differences were found in anxiety scores across time.

Abbreviations: PCI, perceived cognitive impairment; SD, standard deviation.

^a^ Statistically different from T1 with *p* < .05.

^b^ Statistically different from mean T1 score with *p* < .001.

^c^ Lower scores on the PCI subscale of the FACT‐cog are indicative of worse perceived cognitive impairment.

### 4.4 Relationships between change in perceived cognition, insomnia, and fatigue over time

**FIGURE 2:**
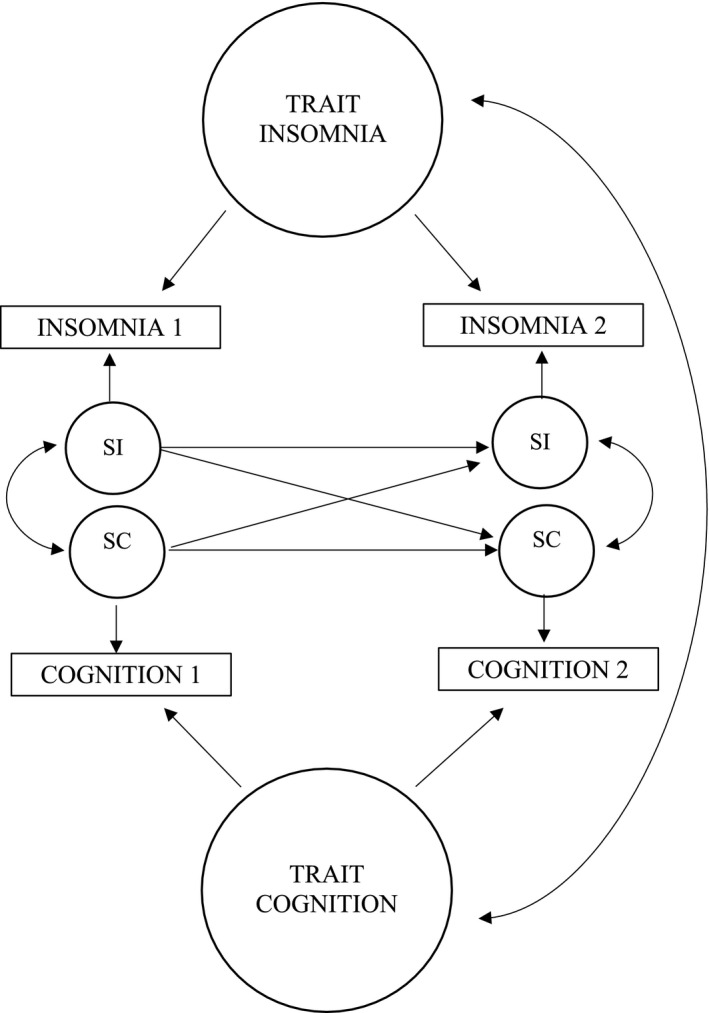
Example of the RI‐CLPM depicting the relationship between two variables—cognition and insomnia—over time. SC, state cognition; SF, state fatigue; SI, state insomnia

The RI‐CLPM evaluating covariation between change in perceived cognition, insomnia, and fatigue over time indicated good model fit, TLI = 0.97, RMSEA = 0.058; for simplification purposes, an example of the RI‐CLPM is illustrated in Figure 2 using two variables. The means, residual variance, and random effect variance estimates for cognition, fatigue, and insomnia and full model figure are included in Table S1 and Figure S1. At the trait‐like level, there was a strong negative association between perceived CI and fatigue (*β* = −0.78, *z* = 17.48, *p* < .01) and symptoms of insomnia (*β* = −0.58, *z* = 5.24, *p* < .01), indicating that women who reported high perceived CI reported high fatigue and symptoms of insomnia. There was also a strong positive trait‐level association between fatigue and symptoms of insomnia (*β* = 0.74, *z* = 9.70, *p* < .01), indicating that women who reported high fatigue reported high symptoms of insomnia.

After accounting for the above trait‐level associations, perceived CI at a preceding timepoint predicted perceived CI at the following measurement (*p* < .05). Neither symptoms of insomnia, nor fatigue predicted future perceived CI. A similar association was found for fatigue, wherein fatigue experienced at one timepoint predicted future reported fatigue (*p* < .01). Neither symptoms of insomnia, nor perceived CI from one time point predicted future reported fatigue. Symptoms of insomnia at one timepoint did not predict symptoms of insomnia at the following measurement. Fatigue (*p* < .001), but not perceived cognitive impairment, was a significant predictor of future reported symptoms of insomnia.

## 5 DISCUSSION

### 5.1 Prevalence of objective cognitive impairment over time

This study is one of the first to investigate objective and perceived CI before and during the first year of treatment for breast cancer. Prevalence of objective CI remained relatively consistent, with estimates ranging from 3.1% to 8.2%. Prior to receiving any treatment, 6.1% of our sample presented with objective CI. Other research has found that women who exhibit pretreatment objective CI do so on only the most challenging attention and memory tests.^33^ While the prevalence of objective CI observed in this study during and after breast cancer treatment is lower than reported in previous studies, it appears to be consistent with prevalence rates observed in healthy, cancer‐free populations.^34^ The discrepancy between the prevalence rates observed in the present study and estimates found in other studies may be attributable to different sample characteristics and/or the lower sample sizes reported in some of the previous research, which would affect the generalizability of the results to the larger population of women with breast cancer. This discrepancy may also be attributable to differences in the definition and measurement of CI.^35^ Specifically, we assessed for objective CI using three objective measures. Thus, it is possible that the lower number of tests included in this study resulted in a more conservative estimate of CI. More research that follows recommended guidelines for assessment of CI is needed to increase confidence in the findings and make more accurate comparisons across studies.

### 5.2 Changes in perceived cognitive impairment, insomnia, fatigue, and mood over time

In addition to a worsening of insomnia, fatigue, and depression, just over one third of the sample reported clinically meaningful declines in their perception of their cognitive functioning that persisted throughout the study duration. When compared to prevalence of objective CI, these results suggest that it is the perception of cognitive functioning that is of greater concern among women undergoing breast cancer treatment. A number of studies on cognition and cancer have reported weak or absent associations between objective and subjective cognition.^36^, ^37^ As such, perceived CI may be a more accurate reflection of the impact of even subtle changes in cognition on functioning and quality of life.^37^, ^38^ Studies with larger samples are required to better understand associations between perceived and objective CI, insomnia, fatigue, and mood disturbance over time.

### 5.3 Relationships between change in cognition, insomnia, and fatigue over time

Results from the structural equation modeling demonstrated that trait‐like symptoms of fatigue, insomnia, and perceived CI endured over the 12‐month study duration. These side effects were also strongly associated, suggesting that women who experience problems in one domain were more likely to report problems in others. After accounting for trait‐like associations, neither insomnia nor fatigue were predictive of later CI, which refuted our initial hypothesis and suggests that recent fluctuations in insomnia and fatigue may have little effect on perceived cognition months later. Short‐term fluctuations in fatigue did, however, predict subsequent fatigue and insomnia. These results, in part, mirror results from previous research indicating that the presence of fatigue earlier in treatment is one of the strongest predictors of posttreatment fatigue.^39^, ^40^, ^41^ Women with breast cancer also have worse performance on measures of processing speed when they report relatively high levels of fatigue.^42^

Prompt intervention may change the trajectory of insomnia, fatigue, and perceived CI over time given that women are experiencing these concerns early on in treatment. Physical activity (both aerobic and resistance/strength training) is considered a category 1 recommendation (i.e., highest level of evidence) for fatigue by the National Comprehensive Cancer Network.^43^ A meta‐analysis of randomized controlled trials demonstrated significant reductions in fatigue levels following exercise interventions.^44^ Physical activity has also proven beneficial for CI, which may be at least partially explained by improvements in symptoms of depression that result from increased behavioral activation.^45^ Further, there is evidence that even low intensity exercises can benefit cognition.^46^ Physicians and their patients would thus benefit from discussions around the incorporation of physical activity into the treatment plan at diagnosis and before beginning treatment.

In the present study, state‐like insomnia did not predict itself over time, suggesting that the trait‐like component represents a more meaningful target for intervention due to its overriding importance over time. Thus, the best interventions for insomnia will be those with enduring long‐term effects (e.g., Cognitive Behaviour Therapy for Insomnia; CBT‐I). CBT‐I has demonstrated immediate and long‐lasting effects for reduction of insomnia severity in cancer patients,^47^ and has been shown to reduce fatigue over time.^48^ As such, CBT‐I might be an important first‐line treatment option for breast cancer patients with a primary insomnia complaint and secondary fatigue symptoms.

## 6 STRENGTHS AND LIMITATIONS

The present study's unique use of objective measures of cognitive function recommended by the ICCTF in combination with an empirically validated subjective cognitive measure increases the confidence in the prevalence estimates observed. This study is also the first to use structural equation modeling to investigate the relationships between perceived CI, insomnia, fatigue and mood throughout the first year of treatment. This statistical method allowed us to explore directionality of associations between symptoms; however, we cannot fully infer causality because of the potential influence of other unmeasured variables. Lastly, the present model is calibrated only to detect fairly slow associations (e.g., the impact that having fatigue a few months prior would have on cognitive abilities today). If more rapid relations existed, we would be unable to detect them using the chosen analysis.

## 7 CONCLUSION & IMPLICATIONS

Women report symptoms of CI, insomnia, and fatigue at cancer diagnosis, and these concerns are associated and remain stable across the first year of treatment. Naturally occurring fluctuations in fatigue and insomnia at any given point in time do not predict future CI symptomatology, suggesting that early identification and targeted interventions are required to bring about meaningful improvements in perceived CI. To address this issue, it would be beneficial to invest in the development of multicomponent interventions that can be effectively tailored to address diverse distressing concerns among breast cancer patients in a manner that is cost‐effective. Future research should focus on the early identification and delivery of interventions for cognitive, psychological, and behavioral concerns in women with breast cancer in order to improve overall recovery and well‐being.

## CONFLICTS OF INTEREST

The authors have no conflicts of interest to disclose.

## AUTHOR CONTRIBUTIONS

Nicole Rodriguez: Conceptualization, formal analysis, funding acquisition, investigation, project administration, writing—original draft, and writing—review and editing. Jonathan Fawcett: Conceptualization, data curation, formal analysis, methodology, software, supervision, and writing—review and editing. Joshua Rash: Conceptualization, formal analysis, supervision, and writing—review and editing. Renee Lester: Resources, supervision, and writing—review and editing. Erin Powell: Resources, supervision, and writing—review and editing. Connor MacMillan: Data Curation, resources, supervision, and writing—review and editing. Sheila Garland: Conceptualization, funding acquisition, investigation, methodology, project administration, resources, supervision, validation, writing—original draft, and writing– review and editing.

## Supporting information

Fig S1

Table S1

## Data Availability

The data sets generated during and/or analyzed during the current study are available from the corresponding author on reasonable request. This research has been reviewed by the Interdisciplinary Committee on Ethics in Human Research and found to be in compliance with Memorial University's Ethics policy.
