# Supplementary material for: Factors associated with cognitive impairment during the first year of treatment for nonmetastatic breast cancer
Source: Cancer Med. 2021 Jan 16;10(4):1191–200. doi: 10.1002/cam4.3715 (PMC7926005; doi:10.1002/cam4.3715)
Supplement: Supplementary file 1 — Fig S1 [file CAM4-10-1191-s001.docx]

**Supplementary Figure**. RI-CLPM Model

Supplementary Figure. **Model depicting the relationship between cognition, fatigue, and insomnia over time.** SI = state insomnia, SC = state cognition, SF = state fatigue. **p*<.05, ***p*<.01

^a^ State cognition represents one variable but is presented twice to allow the reader to better visualize the model

Fit Parameters:

TLI = .97

RMSEA = .058

-.08

1.00

1.00

**-21.13****

**-76.38****

-0.92

**40.20****

**19.33****

2.32

-23.44

**-49.53****

**-5.61****

**.31****

-.24

**.27***

**.11****

.02

.02

.21

**.27***

-.02

1.00

1.00

1.00

1.00

1.00

1.00

1.00

1.00

1.00

1.00

1.00

1.00

FATIGUE 1

FATIGUE 2

COGNITION 2

COGNITION 1

INSOMNIA 2

INSOMNIA 1
