# Supplementary material for: Factors associated with cognitive impairment during the first year of treatment for nonmetastatic breast cancer
Source: Cancer Med. 2021 Jan 16;10(4):1191–200. doi: 10.1002/cam4.3715 (PMC7926005; doi:10.1002/cam4.3715)
Supplement: Supplementary file 2 — Table S1 [file CAM4-10-1191-s002.docx]

**Supplementary table.** Mean, residual variance, and random effect variance estimates for cognition, fatigue, and insomnia

|  |  | **T1** | **T2** | **T3** | **T4** |
| --- | --- | --- | --- | --- | --- |
| PCI | Mean | 56.45 | 52.96 | 52.68 | 52.62 |
|  | Residual Variance | 37.13 | 43.60 | 43.60 | 43.60 |
|  | RI Variance^a^ = 68.58 |  |  |  |  |
| Fatigue | Mean | 5.84 | 12.77 | 13.09 | 10.03 |
|  | Residual Variance | 155.59 | 170.50 | 170.50 | 170.50 |
|  | RI Variance = 148.00 |  |  |  |  |
| Insomnia | Mean | 6.81 | 9.91 | 9.25 | 8.49 |
|  | Residual Variance | 17.67 | 13.83 | 13.83 | 13.83 |
|  | RI Variance = 37.13 |  |  |  |  |

Abbreviations: PCI, perceived cognitive impairment

^a^RI Variance = Variance corresponding to the random effect of each variable

Supplementary Table. **Mean, residual variance, and random effect variance estimates for cognition, fatigue, and insomnia.** Mean and residual variance for each time point and each variable, including the variance corresponding to the random effect of each variable.
